# Whole genome sequencing and genetic variations in several dengue virus type 1 strains from unusual dengue epidemic of 2017 in Vietnam

**DOI:** 10.1186/s12985-020-1280-z

**Published:** 2020-01-20

**Authors:** Thuy Thi Dang, My Ha Pham, Huy Vu Bui, Duyet Van Le

**Affiliations:** 1 National Hospital for Tropical Diseases, 78 Giai Phong, Dong Da, Hanoi, Vietnam; 2 National Hospital for Tropical Diseases Oxford University Clinical Research Unit, 78 Giai Phong, Dong Da, Hanoi, Vietnam; 3 Hanoi Medical University, 1st Ton That Tung, Dong Da, Hanoi, Vietnam

**Keywords:** Dengue virus (DENV), Full-length genome, Dengue virus type 1 (DENV-1), Genetic variants, Dengue epidemic

## Abstract

**Background:**

Dengue hemorrhagic fever is an acute viral infection transmitted by mosquitoes. In the 2017, a dengue epidemic occurred in Hanoi in a short time interval and many cases were serious with associated mortality. This was the largest and unusual dengue fever outbreak in the North of Vietnam over the past 20 years. The objective of the present study was to understand the genetic characteristics of the DENV-1 strain in the 2017 epidemic and its relationship with previous viruses in Vietnam and the rest of the world.

**Methods:**

Complete genomes of 72 DENV-1 from patients in the 2017 epidemic were sequenced using NGS. The full genome sequences were then analyzed to find out the genetic variants in the groups of 72 strains, followed by their comparison with other strains that caused disease in Vietnam previously and several other regions of the world, revealing a genetic relationship between them.

**Results:**

The complete genome sequence of 72 DENV-1 strains comprised 10,697 nucleotides with an open reading frame coding for 3392 amino acids. The genomic analysis revealed different amino acid substitutions in all genes, especially varying at position S75 (Capsid), M125 (PrM), D54 (E), T147, V180 (NS1), G45, Y126, I154 (NS2A), A94 (NS2B), M298 (NS3), K47, V68 (NS4A), I29 (NS4B), and R166, E536, G614, T821 (NS5). The genetic analysis suggested that the viruses were most closely related to the causative virus of the dengue outbreak in Vietnam and Cambodia from 2006 to 2008. These results indicated that DENV-1 from the dengue epidemic 2017 in Northern Vietnam originated from the virus that caused the dengue outbreak during the 2007 to 2008 period in Vietnam.

**Conclusion:**

The present study is the first of its kind to describe complete genome sequence as well as genetic variants and phylogenetic analysis of DENV-1 associated with the unusual dengue epidemic of 2017 in northern Vietnam. These results provide detailed evidence to elucidate the origin, circulation, and genetic evolution of DENV in Vietnam.

## Background

Dengue hemorrhagic fever is a mosquito-borne acute infection caused by one of four dengue viruses (DENV), the disease mainly spread in tropical and subtropical countries. The virus is transmitted to humans via the bite of an infected mosquito [1]. Clinical manifestations primarily include acute fever, accompanied by bleeding under the skin or mucosa and thrombocytopenia, which may be fatal if not diagnosed and treated promptly and effectively [2]. Dengue hemorrhagic fever can result in major epidemics; these often occur in the Asia and Western Pacific countries such as India, Malaysia, Singapore, Thailand, and Vietnam with a disease cycle of 3 to 5 years [3, 4].

There are four dengue viruses, denoted as DENV–1, DENV–2, DENV–3, and DENV–4 [5]. The genome structure of DENV has positive sense RNA genome of 10.6 to 11.0 kb [6]. The genome encodes for a single open reading frame (~ 3400 codons) and is flanked by 5′ UTR and 3′ UTR (untranslated region) [7]. The genome encodes for three structural proteins, namely C protein, M protein, and E protein, and seven non-structural proteins, namely NS1, NS2a, NS2b, NS3, NS4a, NS4b, and NS5 [7]. There are four commonly circulating dengue viruses in the world, which share a similarity of 65 to 70% of amino acid sequences [8].

Genome sequence of DENV-1 are classified into 5 genotypes, including I, II, II, IV and V [9, 10]. Phylogenetic studies have indicated anassociation between specific genotypes and the proportion of cases with more severe disease [4, 11]. Thus, DENV is classified as being of low, medium and high epidemiological impact. In which, several DENV may be retained in sylvatic cycles with little transmissibility to humans, other DENVs cause dengue fever only [4, 11]. In contrast, many genotypes of DENV may be associated with the potential to also cause the more severe dengue hemorrhagic fever and dengue shock syndrome [4, 11].

DENV has been known for over 200 years [12]. The first outbreaks caused by this virus were reported in 1779 in Jakarta (Indonesia) and Cairo (Egypt), and in 1780 in Philadelphia (USA) [13]. Subsequent outbreaks have been recorded in different countries and regions around the world [13]. The disease caused by DENV is most often benign, non-fatal, with main manifestations of high fever and osteoarthritis [14]. However, cases of severe progression and death have also been reported [4]; therefore, dengue is classified as one of the most important diseases transmitted by mosquitoes, of which *Aedes aegypti* is the main vector. According to the World Health Organization (WHO) report of 2009, the countries most severely affected by this disease include those in South and Southeast Asia, and countries in the Caribbean, Central, and South America [15].

Over the past 50 years, the number of global dengue outbreaks has increased 30 fold owing to its spread to several new territories and its transition from urban to rural areas [16]. It is estimated that approximately 2.5 billion people live in tepidemic areas and that around 50 million cases occur per year [17]. The rise of DENV accompanied by increasing severity is thought to have occurred owing to the ever-growing human population, the speed of urbanization, migration, and lack of control measures for mosquitoes, as well as poor health infrastructure in most affected countries.

Dengue in Vietnam has a year-round occurrence, usually increasing in the rainy season with peak numbers of cases observed between June and October [18]. The disease occurs in both children and adults with an increasing number of cases and associated complications. The main factors responsible for the persistance of dengue in Vietnam and the region are high density and widespread distribution of vectors, and the circulation of all four types of DENV [19]. The habit of hoarding domestic water at home or sanitation does not guarantee the ideal environment for vectors such as *A. aegypti* mosquito, and effective control of this mosquito species is not yet available.

In Vietnam from 2001 to 2010, the total number of cases reported in 19 provinces was 592,938 [20]. Hanoi is one of the two biggest cities located in the North of Vietnam. In the previous large outbreak in 2009 with 16,263 cases were recorded that spread to all districts and Hanoi recorded 87% of all patients. The number of people infected with dengue in 2009 is 6.7 times higher than in 2008 [21]. In 2014, Vietnam recorded 43,000 cases in 53 provinces with 28 deaths [22]. The unusual epidemic outbreak in 2017 did not occur in the peak season of the disease; however, it reported a high number of cases (183,287 cases and 154,552 hospitalizations) in a short period (from June to August 2017) and occurred in all age groups, ranging from young children, the elderly, adolescents, and adults, to pregnant women [23]. In this outbreak, there were 59,063 dengue fever cases in Northern Vietnam, approximately eight times higher than in 2016 with 7289 dengue fever cases [24]. In this ourbreak in Hanoi, the number of cases was 70 times higher compared to the same period in 2016, with nearly 37,651 people infected and seven deaths [25].. DENV-1 accounted for the largest proportion of detected viruses in the 2017 epidemic besides types 2, 3, and 4 [26]. Previous genetic studies in Vietnam on this virus involved only analysis of the E-gene sequence [27–30]. Thus, not much information is available on the genetic diversity of the whole DENV-1 genome over time and the genetic diversity of the DENV resulting due to synonymous and non-synonymous mutations that make the DENV adaptable under selective pressure. Moreover, the construction of the phylogenetic tree of the causative strains of the unusual epidemic in 2017 required the dataset at a global level.

Here, we sequenced the complete genome of 72 DENV-1 strains circulating in the unusual outbreak of dengue hemorrhagic fever 2017 in Northern Vietnam. We also analyzed the genetic variants and genetic relationships of these strains with others that circulated in Vietnam as well as in Southeast Asia and around the world previously. We believe the data obtained from this research will provide significant evidence to enhance our knowledge on the circulation and genetic evolutionary characteristics of DENV in Vietnam.

## Methods

### Patients and samples setting

A total of 300 adult patients presenting at the National Hospital of Tropical Diseases in Hanoi with clinical symptoms of dengue (fever, fatigue, and muscle and joint aches) within three days of symptom onset, and a positive NS-1 test were included in the study between June and August 2017. Patients were tested for NS1 antigen using a rapid test kit from Cortez (USA). Blood samples (2–3 mL) of patients with acute dengue fever were collected and centrifuged. The plasma was separated and stored at − 80 °C. All patients signed written informed consent forms to participate in the study. This study was reviewed and approved by ethical board of the National Hospital of Tropical Diseases.

### Dengue serotyping and quantification

DENV were typed and quantified using Real-time One-Step Reverse Transcription PCR (RT-PCR). Viral RNA was isolated directly from plasma of NS1-positive patients using Qiagen kits (QIAamp Viral RNA Mini Kit; Qiagen Sciences Germantown, MD, USA) in accordance with the manufacturer’s protocol. Briefly, 140 μL plasma was resuspended in 560 μL of lysis buffer (buffer AVL) containing carrier RNA and was incubated at room temperature for 10 min. Thereafter, 560 μL ethanol (96–100%) was added and the sample was treated in accordance with the manufacturer’s protocol (for microfuge-scale preparations).

The Real-time OneStep Multiplex RT-PCR amplification was performed in a 25-μL reaction volume using SuperScript™ III One-Step RT-PCR system with Platinum™ *Taq* DNA polymerase (Invitrogen; CA, USA). A PCR mix of two reactions was prepared in a 0.2-mL, thin-walled tube; reaction 1 contained 25 μL 2× reaction mix, 1 μL enzyme, 20 pmol of dengue type 2 primers (DENV2-F, DENV2-R), 20 pmol of dengue primers type 4 (DENV4-F, DENV4-R), 10 pmol dengue 2 probe (DENV2-Probe), 10 pmol dengue 4 probe (DENV4-Probe), and 10 ng template ARN. The final volume was made up to 50 μL with distilled water. The reaction 2 contained 25 μL 2× reaction mix, 1 μL enzyme, 20 pmol of DENV-1 primers (DENV1-F, DENV1-R), 20 pmol dengue type 3 primers (DENV3-F, DENV3-R), 10 pmol of dengue 1 probe (DENV1-Probe), 10 pmol of dengue 3 probe (DENV3-Probe), and 10 ng template ARN. The final volume was made up to 50 μL with distilled water. The primer sequences were listed in Table 1. For viral quantification, four standard positive controls (the concentrations are 10^2^, 10^4^, 10^6^, 10^8^ copies/ml) were added. The PCR was performed using the LightCycler 480 (Roche) at 50 °C for 30 min (cDNA synthesis). The application parameters included 1 cycle of denaturation at 95 °C for 2 min and 45 cycles of initial heat activation at 95 °C for 15 s, annealing at 60 °C for 30 s, followed by reading the fluorescence. The DENV serotypes were determined by specific fluorescence and viral load were calculated via threshold cycle values (Ct).

**Table 1:** Primer sequences used for serotyping and viral load quantification. All primers are new for this work. Primers for each serotype share annealing temperatures, which enables interchangeable use within each set

Primer name	Sequence
DENV1-F	5′ – ATCCATGCCCAYCACCAAT – 3’
DENV1-R	5′ – TGTGGGTTTTGTCCTCCATC – 3’
DENV1-Probe	5′ – FAM – TCAGTGTGGAATAGGGTTTGGATAGAGGAA – 3′ – BHQ
DENV2-F	5′ – TCCATACACGCCAAACATGAA – 3′
DENV2-R	5′ – GGGATTTCCTCCCATGATTCC – 3′
DENV2-Probe	5′ – FAM – AGGGTGTGGATTCGAGAAAACCCATGG – 3′ – BHQ
DENV3-F	5′ – TTTCTGCTCCCACCACTTTC – 3’
DENV3-R	5′ – CCATCCYGCTCCTTGAGA – 3’
DENV3-Probe	5′ – CYAN500 – AAGAAAGTTGGTAGTTCCCTGCAGACCCA – 3′ – BHQ
DENV4-F	5′ – GYGTGGTGAAGCCTCTRGAT – 3’
DENV4-R	5′ – AGTGARCGGCCATCCTTCAT – 3’
DENV4-	5′ – CYAN500 –
Probe	ACTTCCCTCCTCTTYTTGAACGACATGGGA – 3′ – BHQ

### Whole-genome sequencing

Qiagen kits (QIAamp Viral RNA Mini Kit; Qiagen Sciences Germantown, MD, USA) were used for extracting viral RNA from DENV-1 strains (large scale) as described above. The RNA was used as a template to synthesize cDNA (using ProtoScript® II First Strand cDNA Synthesis Kit – New England BioLabs, MA, USA), followed by DNA replication using two primer pools (Additional file 1: Table S1). PCR products were visualized using gel electrophoresis; their concentrations were measured and standardized. The PCR products were cleaved into short DNA fragments, followed by insertion of Index1 and Index2 in accordance with the manufacturer’s protocol. The purification step discarded the excess products. The sample was standardized and quantified to prepare the library. After completing the library preparation, the samples were placed into the Illumina MiSeq machine to read the sequences. The results were then processed to remove the links and poor quality reads from the raw data to obtain the best sequences. The completed sequence of DENV type 1 was then assembled from the reads using the CLC software. We used a DENV-1 sequence from 1997 (GenBank accession number NC_001477.1) as the reference sequence. The coverage of each nucleotide position on the gene sequence was calculated using the SAM tools.

### Genetic variation analysis

The amino acid sequences of Capsid, PrM, E, NS1, NS2A, NS2B, NS3, NS4A, NS4B, and NS5 of DENV-1 circulating in Vietnam (2005, 2008), Thailand (2010), Myanmar (2002), Brunei (2006), Cambodia (2008), China (2017), and Brazil (2010) were obtained from the GenBank nucleotide sequence database with accession numbers FJ882570, KF955446, HG316481, AY726553, GU131922, EU179861, MF681693, and JX669462, respectively. The target regions of the amino acid sequences were analyzed for all 72 DENV-1 sequences using MEGA (version 6.06) and ClustalX 2.1 to find alterations.

### Fast genome distance estimation

Genetic similarities between 72 DENV-1 sequences and 47 reference sequences (the references selection were based on close geographical distance and far geographical distance from Vietnam) were calculated using the MASH 2.0 method in the following two steps: The first step was to create a sketch. For group 1 that consisted of 72 samples of this study, each sequence was cut into short sequences (k-mer) of 20 bp and assigned a random identifier (also known as a hash). Subsequently, MASH selected a set of 1000 k-mer groups (equivalent to 1000 hash groups) that represented the entire genetic sequence of each virus. Thus, the first sketch file included 72 k-mer sets. The above procedure was repeated with group 2 of 47 reference samples to create a second sketch file. The second step included calculating the distance between gene sequences. Once two sketch files were generated, the software compared the set of 1000 hash groups of each sample in group 1 with a set of 1000 hash groups of each sample in group 2. The distance ≤0.5 correlates to average nucleotide identity (ANI) ≥ 95%, whereas the ANI is an indicator of genome similarity at the nucleotide level between the coding regions of two genomes.

### Phylogenetic analysis

The DENV-1 sequences of the present study and 47 DENV-1 references were compared using MAFFT (Multiple Alignment using Fast Fourier Transform; https://mafft.cbrc.jp/alignment/software/). Following this, all single nucleotide polymorphisms (SNPs) from multiple alignment data were separated by SNP-sites method (https://github.com/sanger-pathogens/snp-sites#usage). The IQ-tree system (http://www.iqtree.org) was used to create a phylogenetic tree from the input of SNPs data. Maximum likelihood method and repeatability ultrafast bootstrap 1000 were selected to increase accuracy.

## Results

### Serotyping and quantification

The Real-time RT-PCR results from 300 NS-1 positive dengue patients showed 276 samples to be positive for DENV–1, 22 samples for DENV–2, one sample for DENV–3, and one sample for DENV–4, there were no co-infections detected (Table 2). Viral load quantification revealed that out of a total of 276 DENV–1 samples, only four showed a viral load ≤10^3^ copies/mL, whereas the remaining samples exhibited a load > 10^3^ copies/mL. All 22 DENV–2 samples and two samples of DENV–3 and DENV–4 showed a viral load > 10^3^ copies/mL (Table 2). We selected 72 type 1 dengue samples with the highest viral load to conduct whole-genome sequencing.

**Table 2:** Serotype distribution and viral load of NS1 positive patients

	Number of specimens	Percent (%)
Serotype		
Type 1	276	92.0
Type 2	22	7.3
Type 3	1	0.33
Type 4	1	0.33
Viral load (copies/mL)		
Serotype 1		
≤ 10^3^	4	1.45
> 10^3^	272	98.55
Serotype 2		
≤ 10^3^	0	0
> 10^3^	22	100
Serotype 3		
≤ 10^3^	0	0
> 10^3^	1	100
Serotype 4		
≤ 10^3^	0	0
> 10^3^	1	100

### Full-length sequence and genetic variants

The sequencing results of the 72 DENV1 virus are shown in Additional file 2: Figure S1. The average coverage of each segment of the gene encodes for 10 highly valuable viral proteins. The smallest value falls to around 300×, whereas the maximum value reaches to more than 20,000×. The gene segment encodes for three structural proteins, especially the E protein, with repeatability above 1000×. This increases accuracy when analyzing small changes in the gene. In addition, the rest of the genome encodes seven non-structural proteins that were completely sequenced. In particular, the gene segment of NS1 plays an important role in the replication of DENV RNA with wide repeatability, ranging from 800 to 8000 × .

Amino acid analysis of 72 viruses revealed considerable amino acid alterations throughout the complete coding region. Additional file 1: Table S2 lists the amino acid replacements found in 10 regions. The analysis results indicated 157 amino acid substitutions between 72 DENV-1 strains and 8 reference strains (as shown by the genetic variant analysis). Of these mutations, the comparison result reported that 72 DENV-1 viruses showed several amino acid replacements appearing in all coding regions, in which the majority of mutations appeared in a small number of population (1–6 of 72 viruses). However, several amino acid alterations were found in a large number of strains such as the position 48 (11A/61 T), 125 (25 M/47I) of PrM region, position 54 (16 N/56D) of E region, position 147 (13 T/59A) of NS1, position 45 (9 T/63A), 67 (32G/40G), 126 (33Y/39H), 154 (12I/60 V) of NS2A, position 94 (32A/40 T) of NS2B, position 298 (13 M/59 V) of NS3, position 16 (11 L/61 M), 47 (16R/56 K), 68 (21 V/51 M) of NS4A, position 166 (13R/59 K), 167 (16Y/56H), 337 (4A/19 V/49I), 609 (12A/60 V), 614 (13G/59E), 821 (32 T/40A) of NS5. In addition, the results showed that some DENV-1 strains possessed multiple amino acid substitutions. The comparison results indicated that the amino acid sequences of 72 strains differed significantly from the DENV1 strain that caused epidemics in Vietnam in 2005, but were very similar to the pandemic strains in the year 2007 and 2008. The similarities also disappeared with the strains that circulated in Cambodia and Thailand.

### Genome distance estimation

The similarities between the gene sequences are shown via three parameters, including Mash-distance, *p*-value, and matching hashes. The lower the distance (Mash-distance), the higher the number of k-mer (matching-hashes) groups, implying a greater level of nucleotide similarity between the 2017 DENV1 genomes and reference genomes. The data of Mash-distance, *p*-value, and matching hashes were presented in the box chart of Additional file 2: Figure S2). The results of comparing similarity between 72 DENV1 with 47 reference samples showed that the DENV1 viruses in the present study were at the lowest distance from the DENV1 in Vietnam in 2007 and 2008 (distance, 0.013–0.017), followed by DENV1 strains circulating in Cambodia in 2006, 2008, and Thailand in 2001 (distance, 0.018–0.021), and other DENV1 strains in Southeast Asia and South Asia (Additional file 2: Figure S2). Several DENV1 strains circulated in distant geographic areas, such as Argentina, Brazil, Mexico, the USA, and Columbia and exhibited a large gap with the DENV1 strains in Vietnam in 2017. Especially, DENV1 strains causing diseases in some neighboring countries, such as China (2006), Indonesia (1998), Brunei (2005, 2006), Malaysia (1972), Singapore (2004, 2013), and Myanmar (1976), revealed very high distances with the strains of the present study.

### The similarity of DENV-1 genome sequence

**Fig. 1:**
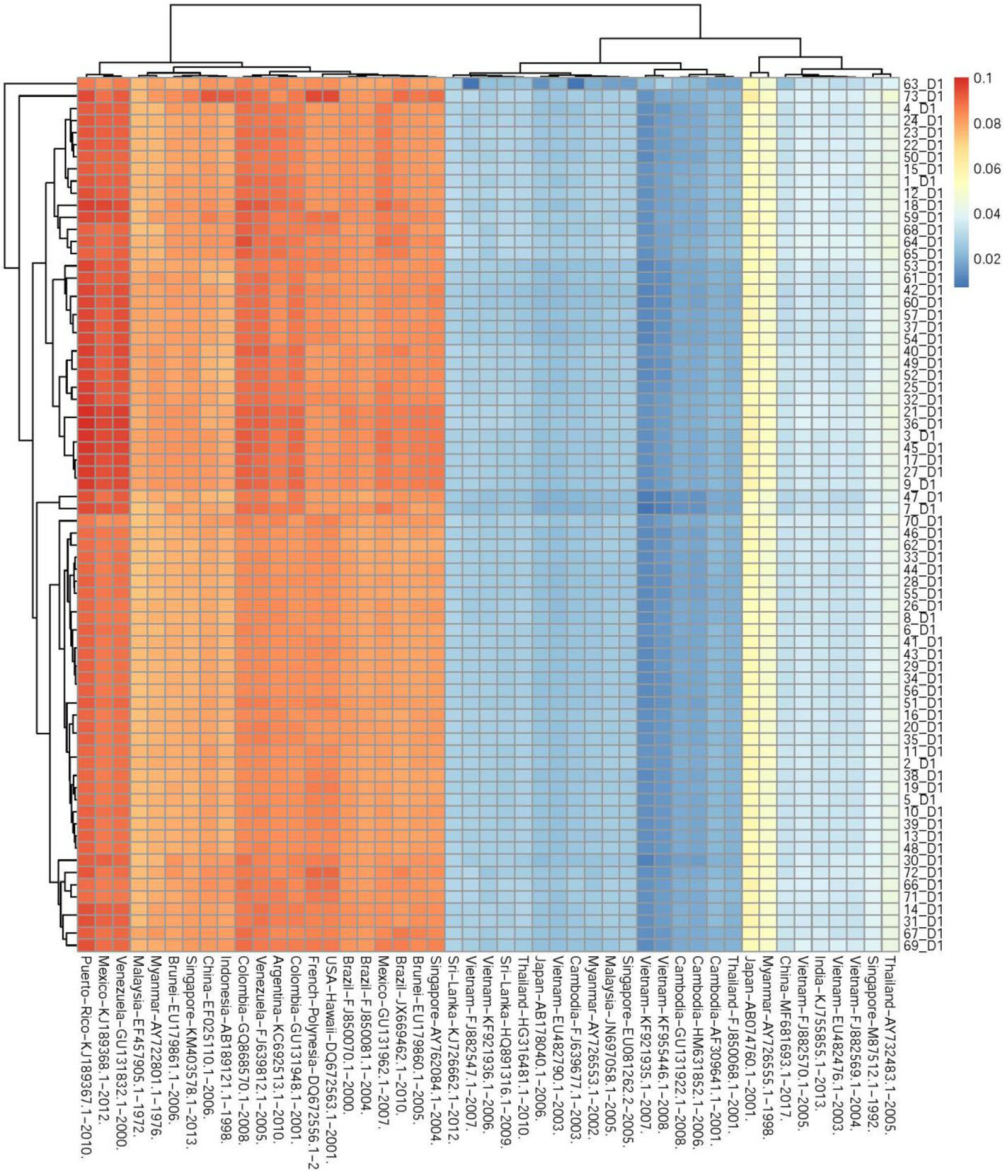
Genomic clustering of 72 DENV–1 samples and 47 reference sequences based on MASH distances. Heatmap illustrates the pairwise similarity between samples, color scale ranges from 0 (blue) to 0.1 (red). Two large groups are identified and colored with the same key. The MASH clustering also reflects the significant difference between references from Asia and from other continents when comparing with the individual samples in this study

Heatmap is a graph that displays information in the form of a matrix, revealing the distance between the two row variables (72 strains of DENV-1) and column (47 dengue reference strains), from cold color to hot color, indicating the gradual increase in the distance value between DENV-1 samples and the dengue reference (Fig. 1). The results of the analysis showed a clear separation into two groups that were being referred to different distance between 2017 DENV1 genomes and reference genomes. Which is geographically reasonable; group 1 of all green means the distance between the samples and reference is small, resulting in a high sequence similarity between the genomes, whereas the orange and red groups showed the inverse result. The DENV1 patterns (denoted D1) revealed a lower similarity to the DENV1 strains from Japan and Myanmar (yellow). Moreover, there seemed to be a significant difference with the strains from Brunei, Indonesia, China, Malaysia and Singapore (orange-red) although these countries are in Southeast Asia. The sequence of D1 samples were also exhibited distinct difference with the strain from American such as Brazil, Colombia, Mexico (red). However, the sequence of D1 samples almost coincided with the pathogen DENV1 that circulated in Vietnam during the 2007–2008 dengue outbreaks (dark blue).

### Phylogenetic tree analysis

**Fig. 2:**
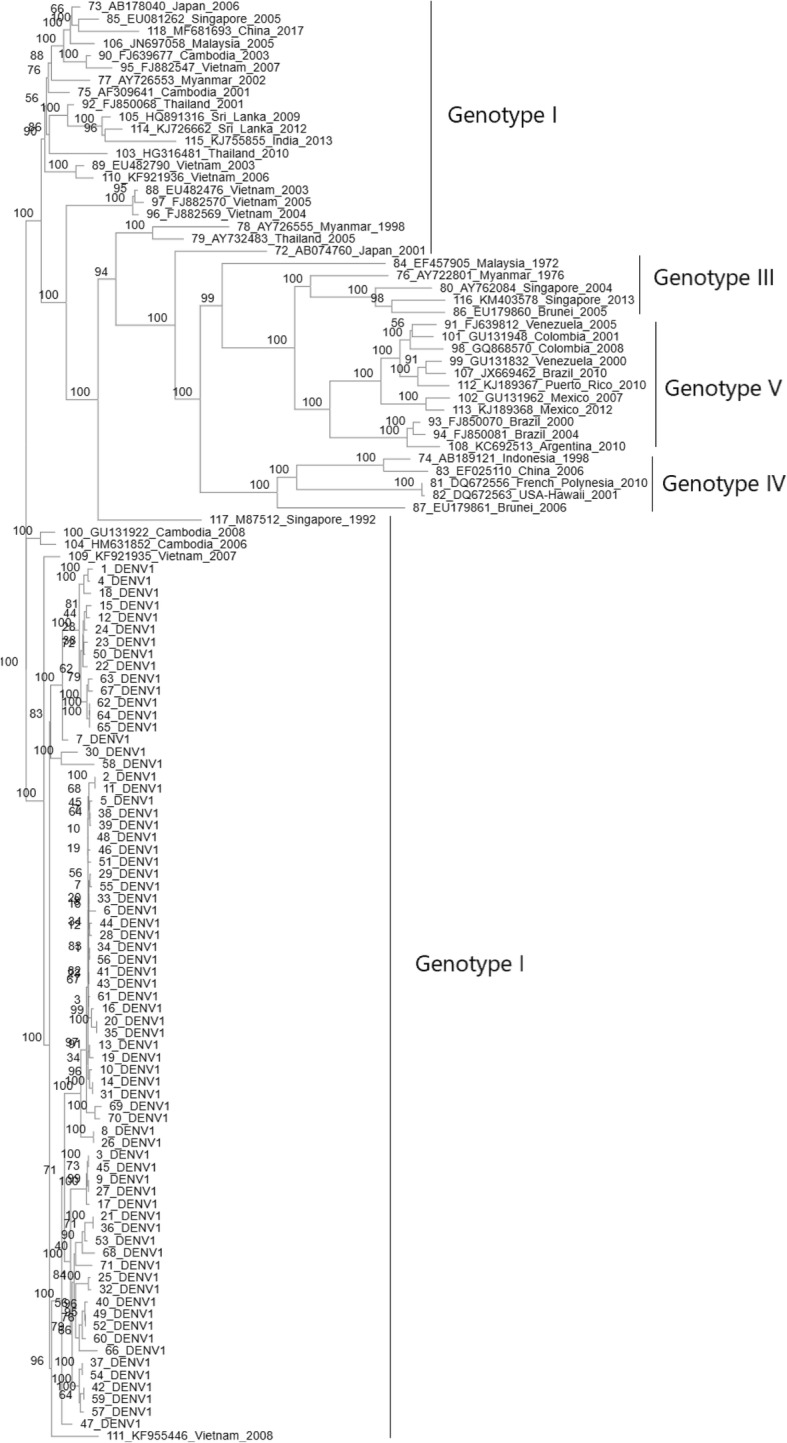
Phylogenetic tree based on the level of a similar sequence of 72 DENV-1 strains and 47 reference viruses. The viruses fromVietnam in 2017 are denoted by D1, the strains circulated in Vietnam in the past and in the world are denoted by the country name, GenBank accession number and the year causing the disease

We next analyzed the genetic relationship between 72 DENV-1 viruses from NS-1 positive dengue patients in Vietnam in 2017 with viruses circulating in Vietnam in previous years and several strains from in other regions of the world. The phylogenetic tree was analyzed and built based on the sequence of the entire viral genome (Fig. 2), in which 72 DENV1 were denoted by 1–72, and reference DENV1 were denoted by 73–119. The analysis results showed that the strains in Vietnam in 2017 had the highest genetic similarity to the strains in Vietnam in 2007 and 2008. In addition, the genetic relationship from phylogenetic tree indicated that these strains belonged to the group of DENV-1 from Vietnam (2007 and 2008), Cambodia (2001, 2006, and 2008), Thailand (2001 and 2010), Myanmar (2002), Malaysia (2005) and were not clustered into the same group with pathogens from USA, Brazil, Mexico, Argentina, Venezuela, and Columbia. Although in the same geographical area, the analysis also revealed that the DENV-1 viruses of this study had a significant difference as compared with the DENV1 strains in Singapore (2013), Myanmar (1976), Malaysia (1972), China (2006), Indonesia (1998), and Brunei (2006).

## Discussion

Previous studies involving genome analysis on the DENV revealed that genetic alteration may lead to a change in the properties and characteristics of the virus [31–33]. We aimed to investigate the amino acid substitutions in the genome of the DENV that caused the large and unusual dengue outbreak that occurred in Hanoi in 2017. We enrolled 300 dengue patients who tested positive for NS1 and had clinical dengue. Our results revealed that 92% (276 viruses) of patients were positive with DENV1, 7.3% (22 viruses) were infected with DENV2 and only 0.7% (2 viruses) was infected with DENV3 and 4. Thus, the serotype distribution of DENV in the epidemic of 2017 was different from the dengue outbreaks in previous years in the same area. The stereotype distribution in the dengue outbreak in 2011 was only DENV1 (11,3%), DENV2 (88,7%) [34], and in the outbreak of 2008, the distribution rates for DENV1 and DENV2 were 32 and 30%, respectively [35]. Recent studies have shown that all four stereotypes were present in dengue outbreaks [5, 16, 36, 37]. The distribution of DENV types in each of the different epidemics may have the prevalence of multiple dengue serotypes, but only one serotype is dominant [5, 38–40].

Other studies on the epidemiology of DENV causing outbreaks in Vietnam have been recorded as iendemic strains, but there were still a number of outbreaks that were caused by strains from some other geographical areas. Therefore, studying the genome characteristics of DENV strains will help to trace the origins of the virus strain in 2017 as well as provide more effective prevention. In this study, we selected 72 DENV-1 viruses with the highest viral load to conduct genome sequencing using NGS Illumina MiSeq system. The sequencing results showed that all 72 DENV-1 were successfully sequenced with high reliability, in which all gene segments were repeated from 1000x to 10,000x, the average reads were 5000x, this is a very high level of repetition for the sequencing of a viral RNA genome by the MiSeq system. The advantage of next-generation sequencing (NGS) is that the nucleotide sequence is read repeatedly, thus allowing the detection of minor mutations that occur in the viral genome.

Genome analysis of 72 DENV-1 viruses showed that there were various amino acid alterations in all structural and non-structural genes. The proportion of viruses with amino acid changes is scarce, ranging from 1 to 6 viruses, which focused only on certain positions on the genes. However, it was recognized that there are major strains of DENV1 carrying amino acid mutations in several positions, such as in the E gene with positions 54D/N, 226 T/A, 347I/M/V, 463A/V. The amino acid alterations in the envelope protein help the virus enhance neutrovirulence during pathogenesis. In addition, several positions with amino acid variants were observed in the NS2A, NS2B, NS3, NS4A, NS4B, NS5 genes. These changes may be related to the DENV virulence, but further studies are needed to clarify the influence of the amino acid substitution of DENV and dengue hemorrhagic fever severe.

Studies on the genetic evolution of DENV shows that the average mutation rate of DENV is approximately 7.5 × 10^− 4^ mutations/position/year, this mutation speed compared to the other RNA viruses is relatively high, it is only slower than the HIV and influenza viruses. In nature, DENV exists in two forms, sylvatic and urban. Studies on the genetic evolution of these two forms show that the mutation rate during the urban cycle (dengue causes disease in humans) is faster than sylvatic cycle, and this is also consistent with the level of circulation and virus replication rate in dengue outbreaks worldwide. Not surprisingly, there were many mutations that occurred in the genome of the DENV that caused outbreak in 2017.

Comparison of genetic mutation characteristics among DENV1 viruses in Vietnam in 2017 with those of DENV1 causing the disease in Vietnam in 2005 and 2008 as well as DENV1 strains in Thailand in 2010, Myanmar in 2002, Cambodia in 2008, Brunei in 2006, China in 2017 and Brazil in 2010 showed differences in many amino acid positions in all genes, especially with the DENV1 strain causing outbreak in 2005 in Vietnam, suggesting that DENV1 in Vietnam 2017 did not share the same ancestor as the DENV1 from 2005, although they both caused outbreaks in the same geographic area. Thus, it can be seen that in the same geographical area there exist many different forms of DENV, the emergence of new DENV forms in a geographical area may be due to the spread from other geographic areas due to globalization.

Analysis of the genetic evolution of DENV1 viruses in Vietnam in 2017 with DENV1 strains causing outbreak in Vietnam and around the world previously showed complicated genetic variations. This is probably one of the main causes leading to the unusual dengue outbreak in Vietnam and other countries in the dengue endemic area.. Therefore, in order to clarify the impact of the amino acid mutation in the viral genome related to clinical severity in humans, further studies are needed to determine their role.

Boxplot and heatmap mapping provides a clear picture of the genetic relationship of 72 viruses with strains in Vietnam and in the world. The analytical results suggested that the DENV1 circulating in the 2017 dengue epidemic very similar to the 2007–2008 dengue outbreaks in Vietnam, with a close relationship to strains from Cambodia. Surprisingly, the DENV1 sequenced from other countries in the same geographical areas, such as China, Malaysia, Myanmar, Brunei, Singapore, and Indonesia, revealed a close genetic relationship with strains from Mexico, Brazil, Argentina, Venezuela, and the USA and exhibited large genetic differences with strains in Vietnam. These findings suggest an introduction of genetic transfer or the spread of DENV from one geographic region to another. This, in turn, could result in the mixing of genetic resources of different origins in order to create new variant strains.

Previous studies on the phylogenetic tree construction of DENV often used the nucleotide sequence of E gene. In this study, through successful sequencing of the complete genome of 72 DENV1 viruses, so the phylogenetic tree was established using the entire genome sequence. The analysis showed that all 72 DENV1 viruses that circulated in Vietnam 2017 were likely derived from the DENV-1 strain that caused outbreaks in Vietnam 2007 to 2008 (Fig. 2). This suggested that the DENV1 was responsible for the outbreak of dengue in Vietnam in 2017 and originated from the DENV that was previously distributed in Vietnam. In addition, the phylogenetic analysis revealed a close relationship between these DENV1 strains and two other DENV type 1 strains from Cambodia (2006 and 2008). These findings confirmed the origin and circulation of DENV in Vietnam. There exists no report of any intrusion of DENV from other geographical areas. However, several studies in China, Brunei, and Singapore have shown DENV1 to be imported from distant geographic areas, such as Brazil, the USA and Columbia.

## Conclusion

The present study is the first of its kind to describe the complete genome sequence of 72 DENV type 1 viruses circulating in the unusual dengue outbreak in 2017 in Northern Vietnam and their genome characteristics compared to several DENV type 1 strains distributed in Vietnam and other parts of the world previously. We believe that these results constitute an important database to elucidate the mechanism of dengue hemorrhagic fever, genetic characteristics of the virus, and the development of dengue vaccine in Vietnam.

## Supplementary information

**Additional file 1: Table S1.** DENV-1 Primer for Whole genome Sequencing. All primers are new for this work. Genome location is reported with respect to GenBank isolate JX669464. The primers share annealing temperatures, which enables interchangeable use within two sets of primers. **Table S2.** Genetic variations in the Dengue type 1 genome circulated in Unusual Dengue Epidemic of 2017 in Vietnam. Description of amino acid substitution in the 72 DENV1 virus compared to the 8 reference strains. Homologous amino acids are denoted by (−), and the modified amino acids are denoted by letters. The amino acid site including replacement of hydrophilic and hydrophobic are listed in each gene.

**Additional file 2: Figure S1.** Representative depth of coverage plots for DENV1 circulated in Vietnam 2017. The 72 DENV1 virus were sequenced on the MiSeq platform at a high depth of coverage. The smallest value falls to around 300x and the maximum value reaches to more than 20.000x. **Figure S2.** The distance estimation between Dengue type 1 samples and references. The box chart depicts the distribution position of the data (respectively from bottom to top). On each stripe, there is a box that represents the overall distance of each sample in the whole set of D1 samples with each of 47 references. The distance between Dengue type 1 sample and references was measure by Mash-distance.

## Data Availability

The data that support the findings of this study are available from the corresponding author upon reasonable request.

## Abbreviations

DENV1: DENV type 1
MAFFT: Multiple Alignment using Fast Fourier Transform
MASH: Matching-hashes
NGS: Next-generation sequencing
