# Supplementary material for: Whole genome sequencing and genetic variations in several dengue virus type 1 strains from unusual dengue epidemic of 2017 in Vietnam
Source: Virol J. 2020 Jan 20;17:7. doi: 10.1186/s12985-020-1280-z (PMC6971860; doi:10.1186/s12985-020-1280-z)
Supplement: Supplementary file 1 — Additional file 1: Table S1. DENV-1 Primer for Whole genome Sequencing. All primers are new for this work. Genome location is reported with respect to GenBank isolate JX669464. The primers share annealing temperatures, which enables interchangeable use within two sets of primers. Table S2. Genetic variations in the Dengue type 1 genome circulated in Unusual Dengue Epidemic of 2017 in Vietnam. Description of amino acid substitution in the 72 DENV1 virus compared to the 8 reference strains. Homologous amino acids are denoted by (−), and the modified amino acids are denoted by letters. The amino acid site including replacement of hydrophilic and hydrophobic are listed in each gene. [file 12985_2020_1280_MOESM1_ESM.docx]

# Table S1: DENV-1 Primer for Whole genome Sequencing.

# All primers are new for this work. Genome location is reported with respect to GenBank isolate JX669464. The primers share annealing temperatures, which enables interchangeable use within two sets of primers.

| **Forward Primer Name** | **Forward Primer Sequence** | **Reverse Primer Name** | **Reverse Primer Sequence** |
| --- | --- | --- | --- |
| DV1-400bp-F1 | GTTGTTAGTCTAYGTGGACCG | DV1-400bp-R1 | AACAAGAGTGACTTTCCTCTTTC |
| DV1-400bp-F3 | GCGATGGAYTTGGGAGAG | DV1-400bp-R3 | CTATTCCCACGCATCGCAT |
| DV1-400bp-F5 | GCAAACTGTGCATTGAAGC | DV1-400bp-R5 | CCATTGTTTGTGGACTAGCCA |
| DV1-400bp-F7 | GTCATATGTGATGTGCACAGG | DV1-400bp-R7 | TATTCCTATTTTCATGGTCCAGGA |
| DV1-400bp-F9 | ACAAATTTCAAGCTGAYTCCCC | DV1-400bp-R9 | TCCTTGATGGCAGCTGACAT |
| DV1-400bp-F11 | CACTCTATGGAGYAATGGAGT | DV1-400bp-R11 | CCATCTGGATCTCATCACYTC |
| DV1-400bp-F13 | GCATCATGGTYGGAGCYAA | DV1-400bp-R13 | CGGAAGCCATGTTGTTTTTTG |
| DV1-400bp-F15 | TCCCAAAAAACAACRTGGCT | DV1-400bp-R15 | GGTCGCTGGTATTGATAATGG |
| DV1-400bp-F17 | TCCAAGATGATGGAACCATGA | DV1-400bp-R17 | ATCACCTGCACTTCTTCTCC |
| DV1-400bp-F19-Ver2 | CATCYGGATCTCCYATYGTGAA | DV1-400bp-R19-Ver2 | AARATTGCAGCYGCYTCACCC |
| DV1-400bp-F21 | CCTTTCCACAGAGYAAYGC | DV1-400bp-R21 | TTGTTTAAAGGCTGTCCCATGTA |
| DV1-400bp-F23 | GTGGAGCTCATGAGAAGAGG | DV1-400bp-R23 | TCTGGCAGTTCTTCCATTGC |
| DV1-400bp-F25 | GCTGGCATATGTGGTGATAGG | DV1-400bp-R25 | CTTGCAGTCCAGGTCCAATTA |
| DV1-400bp-F27 | GATGCGGACYACATGGGC | DV1-400bp-R27 | TAGTATGACCAGCCACCTCT |
| DV1-400bp-F29 | GATGGTGGARCCATGGCT | DV1-400bp-R29 | CATATGATCCATGATAGGCCCA |
| DV1-400bp-F31 | AAGTTGACACRCGCACACC | DV1-400bp-R31 | TCCACTCCGCTGAGTGAATT |
| DV1-400bp-F33 | TGGTAAGGGTRCARAGACCA | DV1-400bp-R33 | TTTCCCTCCCATCCTTCATAAT |
| DV1-400bp-F35 | GTCATATGCACAAATGTGGCA | DV1-400bp-R35 | ACATCCCCACGATGGAGC |
| DV1-400bp-F2 | TACCCCCAACAGCAGGART | DV1-400bp-R2 | ATGTYCCATARGTCACCCA |
| DV1-400bp-F4 | CCYTGAGACACCCAGGATT | DV1-400bp-R4 | GAAGAGCCCACAGCCATT |
| DV1-400bp-F6 | AGCTGACCGACTACGGAG | DV1-400bp-R6 | ATGGTGCGTCTGTTCCTTC |
| DV1-400bp-F8 | AGACACCGCATGGGACTT | DV1-400bp-R8 | CATGATGTTCTCGAGACGAGT |
| DV1-400bp-F10 | ACCTTCATCATYGAYGGCC | DV1-400bp-R10 | ACTGTGGTACCYTCACAYA |
| DV1-400bp-F12 | GGAGARGATGGGTGYTGGTA | DV1-400bp-R12 | CAAGTCCATCCCCCAGCTC |
| DV1-400bp-F14 | TTCCTTGGAGGAGCTGGG | DV1-400bp-R14 | CGGCTGAGCTTCCAGATATAAC |
| DV1-400bp-F16 | GCTGGAGGCATGYTRATAGC | DV1-400bp-R16 | CTGGTGACRTGCCACATTGT |
| DV1-400bp-F18 | AGRGGAGCTGTNCTYATGTA | DV1-400bp-R18 | GTTTTCCCYGATCCTGGRTG |
| DV1-400bp-F20 | TTCCGAAATGGCAGAGGC | DV1-400bp-R20 | CACCCGTTTCCCATTCTTTCT |
| DV1-400bp-F22 | AAAGATGGTCCAGAGCGYG | DV1-400bp-R22 | GGTTGTTCCTTTCCCCRTC |
| DV1-400bp-F24 | ATAGGGAAGCTTCCACAACAC | DV1-400bp-R24 | CAGTCCCATCTCATTGGCTG |
| DV1-400bp-F26 | TTGACAAAGGATGGCCAATATC | DV1-400bp-R26 | TTTGCCATGGAKACYGCTAT |
| DV1-400bp-F28 | CGCTAAACTGAGGTGGTTYGT | DV1-400bp-R28 | TTTCGCACTAGCATTCCTCC |
| DV1-400bp-F30 | CTAGATATCATTGGCCAGAGGAT | DV1-400bp-R30 | GCTGAGTTCCATTGATTTTCATCA |
| DV1-400bp-F32 | CGTGCAATATGGTACATGTGG | DV1-400bp-R32 | TTAAGCCATAAGTTCCGACCTG |
| DV1-400bp-F34 | GGGAACCTTCAAAAGGATGGAA | DV1-400bp-R34 | ACCATTGGTCTTCCCTTTTYCC |
| DV1-400bp-F36 | TACCACCTGGTCRATCCA | DV1-400bp-R36 | CTCACAGGCAGCATAGCT |

**Table S2: Genetic variations in the Dengue type 1 genome circulated in Unusual Dengue Epidemic of 2017 in Vietnam**.

Description of amino acid substitution in the 72 DENV1 virus compared to the 8 reference strains. Homologous amino acids are denoted by (-), and the modified amino acids are denoted by letters. The amino acid site including replacement of hydrophilic and hydrophobic are listed in each gene.

| **AA varians**  **Gen** | **AA position**  **(Protein)** | **72 Dengue strains**  **In this study** | **Vietnam**  **(FJ882570)**  **2005** | **Vietnam**  **(KF955446)**  **2008** | **Thailan**  **(HG316481)**  **2010** | **Myanmar**  **(AY726553)**  **2002** | **Cambodia**  **(GU131922)**  **2008** | **Brunei**  **(EU179861)**  **2006** | **China**  **(MF681693)**  **2017** | **Brazil**  **(JX669462)**  **2010** |
| --- | --- | --- | --- | --- | --- | --- | --- | --- | --- | --- |
| **Capsid** | 26 | **A**(5)/**V**(67) | V | - | - | - | - | - | - | G |
|  | 27 | **P**(2)/**S**(70) | S | - | P | - | - | - | T | - |
|  | 46 | **M**(2)/**L**(70) | L | - | - | - | - | - | - | - |
|  | 75 | **S** (71)/**N**(1) | N | S | - | - | S | - | - | - |
|  | 76 | **R**(2)/**G**(70) | G | - | - | - | - | - | - | - |
|  | 79 | **X**(1)/**K**(71) | K | - | - | - | - | - | - | - |
|  | 86 | **R**(4)/**K**(68) | K | - | - | - | - | - | - | - |
|  | 97 | **K**(1)/**R**(71) | R | - | - | - | - | - | - | - |
|  | 99 | **X**(1)/**K**(71) | K | - | - | - | - | - | - | - |
| **PrM** | 4 | **S**(72) | T | S | S | S | S | S | S | S |
|  | 35 | **D**(5)/**E**(67) | E | - | - | - | - | - | - | - |
|  | 44 | **C**(1)/**R**(71) | R | - | - | - | - | - | - | - |
|  | 48 | **T**(61)/**A**(11) | A | T | - | - | - | T | - | - |
|  | 96 | **A**(7)/**T**(65) | T | - | - | - | - | - | - | - |
|  | 116 | **V**(1)/**A**(71) | A | - | - | - | - | - | - | - |
|  | 125 | **M**(25)/**I**(47) | I | - | T | - | - | - | - | - |
|  | 136 | **T**(2)/**S**(70) | S | - | - | - | - | - | - | - |
| **E** | 54 | **D**(56)/**N**(16) | N | - | - | - | - | - | - | - |
|  | 57 | **I**(1)/**V**(71) | V | - | - | - | I | - | - | - |
|  | 85 | **V**(72) | M | V | V | V | V | V | V | V |
|  | 158 | **A**(2)/**T**(70) | T | - | - | - | - | - | - | - |
|  | 173 | **T**(72) | S | T | T | T | T | - | T | - |
|  | 212 | **R**(2)/**K**(70) | K | - | - | - | - | - | - | - |
|  | 226 | **T**(4)/**A**(68) | A | - | - | - | - | - | - | - |
|  | 227 | **T**(1)/**S**(71) | S | - | - | - | - | - | - | - |
|  | 229 | **L**(4)/**S**(68) | S | - | - | - | - | - | - | - |
|  | 314 | **L**(71)/**V**(1) | V | L | - | - | - | - | - | - |
|  | 322 | **I**(3)/**V**(69) | V | - | - | - | - | - | - | - |
|  | 340 | **L**(1)/**S**(71) | S | - | - | - | - | - | - | - |
|  | 344 | **E**(72) | G | E | E | E | E | E | E | E |
|  | 345 | **R**(1)/**K**(71) | K | - | - | - | - | - | - | - |
|  | 347 | **I**(1)/**M**(2)/**V**(69) | V | - | - | - | - | - | - | - |
|  | 362 | **N**(1)/**D**(71) | D | - | - | - | - | - | - | - |
|  | 386 | **G**(1)/**E**(71) | E | - | - | - | - | - | - | - |
|  | 388 | **T**(2)/**A**(70) | A | - | - | - | - | - | - | - |
|  | 402 | **R**(4)/**K**(68) | K | - | - | - | - | - | - | - |
|  | 463 | **A**(5)/**V**(67) | V | - | - | - | - | I | - | I |
|  | 474 | **N**(1)/**S**(71) | S | - | - | - | - | - | - | - |
| **NS1** | 11 | **Y**(2)/**H**(70) | H | - | - | - | - | - | - | - |
|  | 17 | **D**(72) | G | D | D | D | D | D | D | D |
|  | 20 | **L**(1)/**F**(71) | F | - | - | - | - | - | - | - |
|  | 27 | **A**(1)/**V**(71) | V | - | - | - | - | - | - | A |
|  | 28 | **A**(71)/**T**(1) | V | A | A | A | A | N | A | N |
|  | 38 | **V**(5)/**I**(67) | I | - | - | - | - | - | - | - |
|  | 45 | **Y**(5)/**H**(67) | H | Y | Y | Y | Y | - | - | - |
|  | 59 | **R**(1)/**G**(71) | G | - | - | - | - | - | - | - |
|  | 62 | **A**(1)/**V**(71) | V | - | - | - | - | - | - | I |
|  | 96 | **M**(1)/**I**(71) | I | - | - | - | - | - | - | V |
|  | 147 | **T**(13)/**A**(59) | A | - | - | - | - | - | - | - |
|  | 158 | **V**(1)/**I**(71) | I | - | - | V | - | - | - | I |
|  | 180 | **V**(6)/**I**(66) | I | - | - | - | - | - | - | - |
|  | 187 | **H**(1)/**Q**(71) | Q | - | - | - | - | - | - | - |
|  | 206 | **R**(1)/**K**(71) | K | - | - | - | - | - | - | - |
|  | 121 | **N**(1)/**D**(71) | D | - | - | - | - | G | - | - |
|  | 224 | **C**(1)/**H**(71) | H | - | - | - | - | - | - | - |
|  | 228 | **K**(1)/**R**(71) | R | - | - | - | - | - | - | - |
|  | 258 | **R**(72) | K | R | R | R | R | - | R | R |
|  | 272 | **I**(1)/**V**(71) | V | - | - | - | - | - | F | - |
|  | 284 | **A**(1)/**V**(71) | V | - | - | - | - | - | - | - |
| **NS2A** | 21 | **V**(1)/**A**(71) | A | - | - | - | - | - | - | - |
|  | 23 | **L**(1)/**F**(71) | F | - | - | - | - | - | - | - |
|  | 24 | **F**(72) | L | F | F | F | - | - | F | - |
|  | 28 | **V**(4)/**M**(68) | M | - | - | - | - | - | - | - |
|  | 45 | **G**(9)/**A**(63) | A | - | - | - | - | - | - | - |
|  | 51 | **V**(1)/**M**(71) | M | - | - | - | - | - | - | - |
|  | 66 | **V**(2)/**M**(70) | M | - | - | - | - | - | - | - |
|  | 67 | **G**(32)/**R**(40) | R | - | - | - | - | - | - | - |
|  | 76 | **L**(2)/**F**(70) | F | - | - | - | - | - | - | - |
|  | 78 | **K**(1)/**R**(71) | R | - | - | - | - | - | - | - |
|  | 92 | **C**(1)/**S**(71) | S | - | - | - | - | - | - | - |
|  | 125 | **P**(5)/**S**(67) | S | - | - | - | - | - | - | P |
|  | 126 | **Y**(33)/**H**(39) | H | - | - | - | - | - | - | - |
|  | 138 | **V**(1)/**I**(71) | I | - | - | - | - | V | - | V |
|  | 142 | **F**(72) | L | F | F | F | F | - | F | - |
|  | 153 | **V**(1)/**M**(71) | M | - | - | - | - | - | - | - |
|  | 154 | **I**(12)/**V**(60) | V | - | - | - | - | I | - | - |
|  | 157 | **V**(1)/**I**(71) | I | - | - | - | - | - | - | - |
|  | 161 | **L**(1)/**F**(71) | F | - | - | - | - | - | - | - |
|  | 165 | **C**(2)/**L**(70) | L | - | - | - | - | - | - | - |
|  | 193 | **T**(1)/**A**(71) | A | - | T | - | - | T | - | - |
|  | 195 | **S**(1)/**N**(71) | N | - | - | - | - | - | - | - |
|  | 200 | **K**(2)/**R**(70) | R | - | - | - | - | - | - | - |
|  | 201 | **R**(71)/**K**(1) | K | R | - | - | R | - | - | - |
| **NS2B** | 5 | **I**(72) | V | I | I | I | I | I | I | I |
|  | 13 | **F**(5)/**V**(67) | V | - | - | - | - | - | - | - |
|  | 44 | **L**(2)/**S**(70) | S | - | - | - | - | - | - | - |
|  | 67 | **L**(72) | F | L | L | L | L | L | L | L |
|  | 94 | **A**(32)/**T**(40) | T | - | - | - | - | - | - | - |
|  | 98 | **I**(2)/**V**(70) | V | - | - | - | - | I | - | - |
|  | 104 | **C**(1)/**L**(71) | L | - | - | - | - | M | - | M |
| **NS3** | 61 | **R**(1)/**Q**(71) | Q | - | - | - | - | - | - | - |
|  | 65 | **S**(1)/**N**(71) | N | - | - | - | - | - | - | - |
|  | 75 | **L**(1)/**V**(71) | V | - | - | - | - | - | - | - |
|  | 80 | **K**(1)/**N**(71) | N | - | - | - | - | - | - | - |
|  | 87 | **E**(1)/**A**(71) | A | - | - | - | - | - | - | T |
|  | 94 | **S**(1)/**P**(71) | P | - | - | - | - | S | - | - |
|  | 143 | **T**(4)/**A**(68) | A | - | - | - | - | - | - | - |
|  | 144 | **R**(2)/**K**(70) | K | R | - | - | - | - | - | - |
|  | 145 | **V**(2)/**A**(70) | A | - | - | - | - | H | - | - |
|  | 226 | **G**(1)/**E**(71) | E | - | - | - | - | - | - | - |
|  | 298 | **M**(13)/**V**(59) | V | - | - | - | - | M | - | - |
|  | 307 | **I**(1)/**V**(71) | V | - | - | - | - | A | - | - |
|  | 369 | **D**(1)/**E**(71) | E | - | - | - | - | - | - | - |
|  | 495 | **V**(1)/**I**(71) | I | - | - | - | - | - | - | - |
| **NS4A** | 16 | **L**(11)/**M**(61) | M | - | - | L | - | - | - | - |
|  | 47 | **K**(56)/**R**(16) | R | - | - | - | - | - | - | K |
|  | 60 | **I**(2)/**M**(70) | M | - | - | - | - | - | - | T |
|  | 61 | **S**(2)/**A**(70) | A | - | - | - | - | - | - | - |
|  | 68 | **V**(21)/**M**(51) | M | - | - | - | - | - | - | - |
|  | 70 | **N**(3)/**S**(69) | S | - | - | - | - | - | - | - |
| **NS4B** | 19 | **G**(1)/**E**(71) | E | - | - | - | G | - | - | - |
|  | 25 | **A**(72) | T | A | A | S | A | A | - | - |
|  | 28 | **N**(1)/**D**(71) | D | - | - | - | - | - | - | - |
|  | 29 | **I**(5)/**V**(67) | V | - | - | - | - | - | - | - |
| **NS5** | 19 | **K**(72) | R | K | K | K | K | K | K | K |
|  | 65 | **S**(1)/**T**(71) | T | - | - | - | - | - | - | - |
|  | 94 | **Y**(1)/**H**(71) | H | R | - | - | - | - | Y | - |
|  | 102 | **I**(72) | T | I | I | I | I | - | M | M |
|  | 131 | **I**(4)/**V**(68) | V | - | - | - | - | - | - | - |
|  | 166 | **R**(13)/**K**(59) | K | - | - | - | - | - | - | - |
|  | 167 | **Y**(16)/**H**(56) | H | - | - | - | - | - | - | - |
|  | 176 | **F**(2)/**L**(70) | L | - | - | - | - | - | - | - |
|  | 220 | **K**(1)/R(71) | R | - | - | - | - | - | - | - |
|  | 226 | **T**(1)/**A**(71) | A | - | - | - | - | - | - | - |
|  | 232 | **V**(2)/**A**(70) | A | - | - | - | - | - | - | - |
|  | 237 | **I**(2)/**V**(70) | V | - | - | - | - | - | - | - |
|  | 307 | **T**(1)/**I**(71) | I | - | - | - | - | - | - | - |
|  | 337 | **V**(2)/**T**(3)/**A**(67) | A | - | - | - | - | - | - | T |
|  | 345 | **K**(71)/**R**(1) | R | K | - | - | K | - | - | K |
|  | 355 | **R**(1)/**K**(71) | K | - | - | - | - | - | - | - |
|  | 359 | **A**(4)/**V**(19)/**I**(49) | I | - | - | - | - | - | - | - |
|  | 369 | **I**(1)/**V**(71) | V | - | - | - | - | - | - | - |
|  | 399 | **N**(1)/**D**(71) | D | - | - | - | - | - | - | - |
|  | 402 | **H**(72) | Q | H | H | H | H | H | H | H |
|  | 403 | **R**(72) | K | R | R | R | - | R | R | R |
|  | 412 | **R**(1)/**K**(71) | K | - | - | - | - | - | - | - |
|  | 487 | **N**(2)/D (70) | D | - | - | - | - | - | - | - |
|  | 492 | **S**(2)/**P**(70) | P | - | - | - | - | - | - | - |
|  | 518 | **R**(71)/**K**(1) | K | R | - | - | R | - | - | - |
|  | 532 | **K**(72)/ | T | K | K | K | K | - | K | - |
|  | 533 | **A**(1)/**S**(71) | S | - | - | - | - | - | - | - |
|  | 536 | **E**(5)/**K**(67) | K | - | - | - | - | - | - | - |
|  | 596 | **S**(72) | L | S | S | S | S | T | S | - |
|  | 603 | **T**(1)/**P**(71) | P | - | - | - | - | - | - | - |
|  | 609 | **A**(12)/**V**(60) | V | - | - | - | - | - | - | A |
|  | 610 | **F**(3)/**L**(69) | L | - | - | - | - | - | - | - |
|  | 611 | **G**(1)/**D**(71) | D | - | - | - | - | - | - | - |
|  | 614 | **G**(13)/**E**(59) | E | - | - | - | - | - | - | - |
|  | 653 | **I**(2)/**V**(70) | V | - | - | - | - | - | - | - |
|  | 715 | **K**(3)/**R**(69) | R | - | - | - | - | - | - | - |
|  | 751 | **A**(2)/**V**(70) | V | G | - | - | - | - | - | - |
|  | 754 | **I**(71)/**V**(1) | V | I | - | - | I | - | - | - |
|  | 796 | **I**(72) | V | I | I | - | I | - | I | - |
|  | 800 | **E**(6)/**G**(66) | G | - | E | E | - | E | E | E |
|  | 821 | **T**(32)/**A**(40) | A | - | - | - | - | - | - | - |
|  | 827 | **T**(72) | S | T | T | T | T | T | T | T |
|  | 859 | **S**(71)/**T**(1) | G | S | S | S | S | S | S | S |
