# Supplementary material for: Whole genome sequencing and genetic variations in several dengue virus type 1 strains from unusual dengue epidemic of 2017 in Vietnam
Source: Virol J. 2020 Jan 20;17:7. doi: 10.1186/s12985-020-1280-z (PMC6971860; doi:10.1186/s12985-020-1280-z)
Supplement: Supplementary file 2 — Additional file 2: Figure S1. Representative depth of coverage plots for DENV1 circulated in Vietnam 2017. The 72 DENV1 virus were sequenced on the MiSeq platform at a high depth of coverage. The smallest value falls to around 300x and the maximum value reaches to more than 20.000x. Figure S2. The distance estimation between Dengue type 1 samples and references. The box chart depicts the distribution position of the data (respectively from bottom to top). On each stripe, there is a box that represents the overall distance of each sample in the whole set of D1 samples with each of 47 references. The distance between Dengue type 1 sample and references was measure by Mash-distance. [file 12985_2020_1280_MOESM2_ESM.docx]

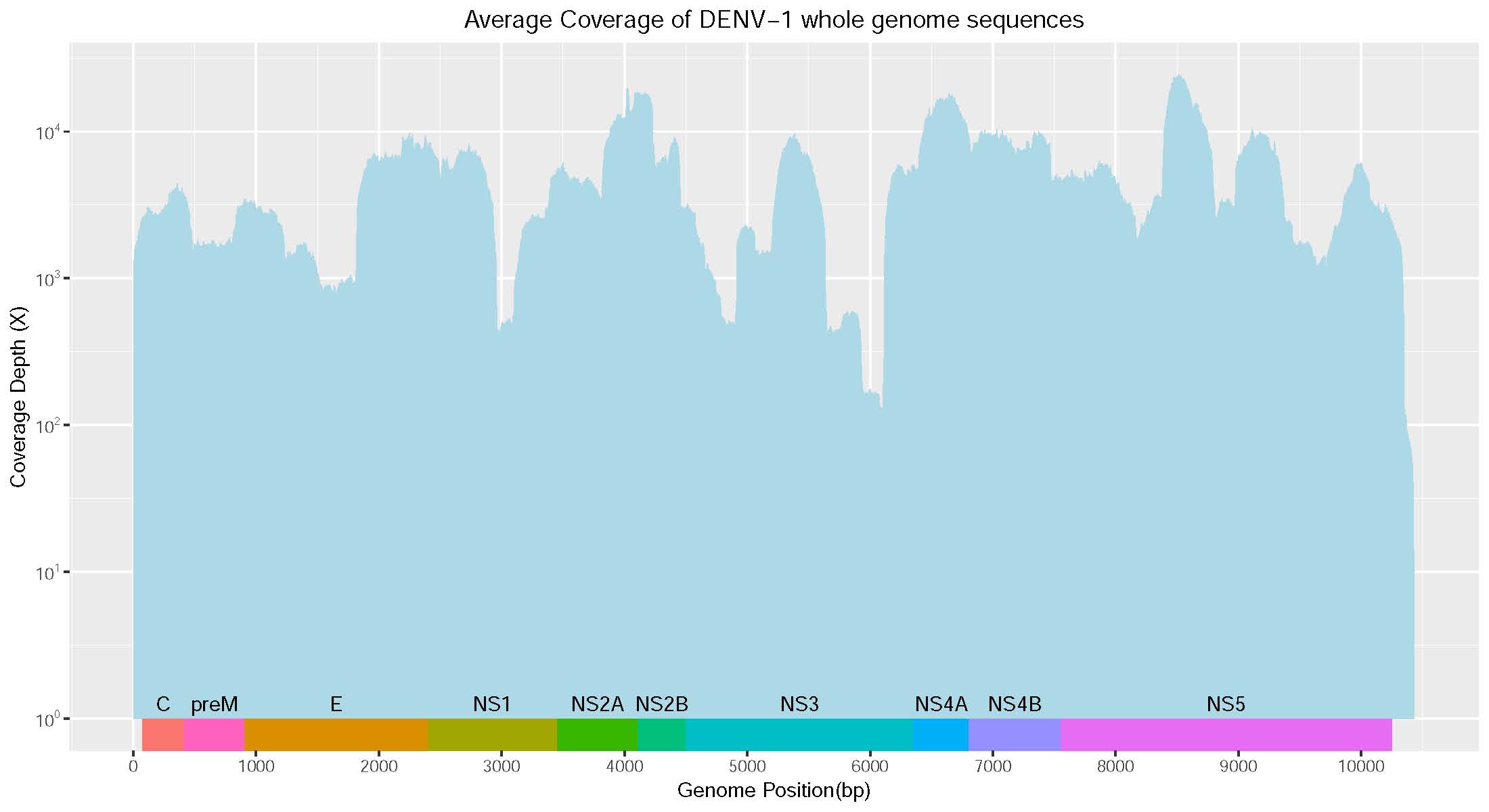


**Figure S1: Representative depth of coverage plots for DENV1 circulated in Vietnam 2017.** The 72 DENV1 virus were sequenced on the MiSeq platform at a high depth of coverage. The smallest value falls to around 300x and the maximum value reaches to more than 20.000x.


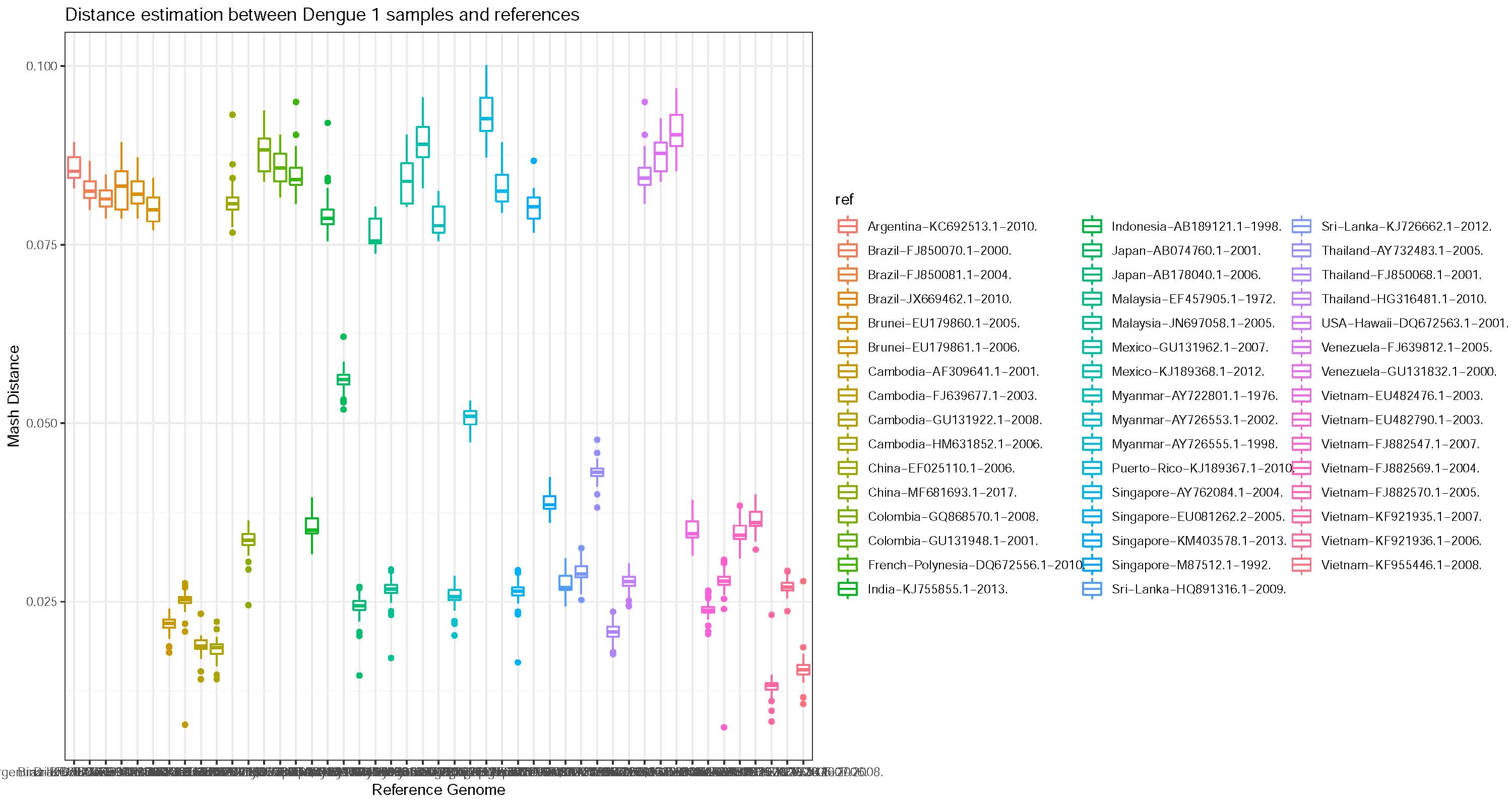


**Figure S2: The distance estimation between Dengue type 1 samples and references**. The box chart depicts the distribution position of the data (respectively from bottom to top). On each stripe, there is a box that represents the overall distance of each sample in the whole set of D1 samples with each of 47 references. The distance between Dengue type 1 sample and references was measure by Mash-distance.
